# Characterization of Wild and Captive Baboon Gut Microbiota and Their Antibiotic Resistomes

**DOI:** 10.1128/mSystems.00016-18

**Published:** 2018-06-26

**Authors:** Pablo Tsukayama, Manish Boolchandani, Sanket Patel, Erica C. Pehrsson, Molly K. Gibson, Kenneth L. Chiou, Clifford J. Jolly, Jeffrey Rogers, Jane E. Phillips-Conroy, Gautam Dantas

**Affiliations:** a Edison Family Center for Genome Sciences and Systems Biology, Washington University in St. Louis School of Medicine, St. Louis, Missouri, USA; b Department of Cellular and Molecular Sciences, Universidad Peruana Cayetano Heredia, Lima, Peru; c Department of Pathology & Immunology, Washington University in St. Louis School of Medicine, St. Louis, Missouri, USA; d Department of Anthropology, Washington University in St. Louis, St. Louis, Missouri, USA; e Department of Anthropology, New York University, New York, New York, USA; f Human Genome Sequencing Center, Baylor College of Medicine, Houston, Texas, USA; g Department of Neuroscience, Washington University in St. Louis School of Medicine, St. Louis, Missouri, USA; h Department of Biomedical Engineering, Washington University in St. Louis, St. Louis, Missouri, USA; i Department of Molecular Microbiology, Washington University in St. Louis School of Medicine, St. Louis, Missouri, USA; University of Colorado Denver

**Keywords:** antibiotics, antimicrobial resistance, baboon, metagenomics, microbial ecology, microbiome, resistome

## Abstract

Environmental microbes have harbored the capacity for antibiotic production for millions of years, spanning the evolution of humans and other vertebrates. However, the industrial-scale use of antibiotics in clinical and agricultural practice over the past century has led to a substantial increase in exposure of these agents to human and environmental microbiota. This perturbation is predicted to alter the ecology of microbial communities and to promote the evolution and transfer of antibiotic resistance (AR) genes. We studied wild and captive baboon populations to understand the effects of exposure to humans and human activities (e.g., antibiotic therapy) on the composition of the primate fecal microbiota and the antibiotic-resistant genes that it collectively harbors (the “resistome”). Using a culture-independent metagenomic approach, we identified functional antibiotic resistance genes in the gut microbiota of wild and captive baboon groups and saw marked variation in microbiota architecture and resistomes across habitats and lifeways. Our results support the view that antibiotic resistance is an ancient feature of gut microbial communities and that sharing habitats with humans may have important effects on the structure and function of the primate microbiota.

**IMPORTANCE** Antibiotic exposure results in acute and persistent shifts in the composition and function of microbial communities associated with vertebrate hosts. However, little is known about the state of these communities in the era before the widespread introduction of antibiotics into clinical and agricultural practice. We characterized the fecal microbiota and antibiotic resistomes of wild and captive baboon populations to understand the effect of human exposure and to understand how the primate microbiota may have been altered during the antibiotic era. We used culture-independent and bioinformatics methods to identify functional resistance genes in the guts of wild and captive baboons and show that exposure to humans is associated with changes in microbiota composition and resistome expansion compared to wild baboon groups. Our results suggest that captivity and lifestyle changes associated with human contact can lead to marked changes in the ecology of primate gut communities.

## Abstract (precis)

Antibiotic exposure results in acute and persistent shifts in the composition and function of microbial communities associated with vertebrate hosts. However, little is known about the state of these communities in the era before the widespread introduction of antibiotics into clinical and agricultural practice. We characterized the fecal microbiota and antibiotic resistomes of wild and captive baboon populations to understand the effect of human exposure and to understand how the primate microbiota may have been altered during the antibiotic era. We used culture-independent and bioinformatics methods to identify functional resistance genes in the guts of wild and captive baboons and show that exposure to humans is associated with changes in microbiota composition and resistome expansion compared to wild baboon groups. Our results suggest that captivity and lifestyle changes associated with human contact can lead to marked changes in the ecology of primate gut communities.

## INTRODUCTION

Antibiotic use in medicine and agriculture has steadily increased in recent decades. This has led to acute and persistent perturbations in bacterial communities in virtually all human-associated environments, including the evolution of multidrug-resistant pathogens that compromise our ability to treat infectious disease (1, 2). In contrast to the recent emergence of clinical resistance in response to antibiotic use, resistance in environmental bacteria is an ancient and prevalent feature of natural ecosystems (3–6). Evidence suggests that environmental microbes have harbored antibiotic production capacities for millions of years and thereby evolved resistance mechanisms on the same time scale to enable self-protection (7). Accordingly, antibiotic-producing microbes likely are the evolutionary progenitor of modern resistance genes and also provided selection pressure for their neighbors to evolve or acquire resistance genes (8). By extension, the microbiota of humans and other vertebrates has likely been exposed to antibiotics produced by environmental bacteria (6) and, concomitantly, their antibiotic resistance (AR) genes before the era of anthropogenic antibiotic use (3, 9). Importantly, the gut microbiota has been shown to be a rich reservoir of AR genes which may be exchanged with pathogens (10–12).

We were interested in how the primate gut microbiota and mobility of its encoded resistome may have been influenced by the modern use of antibiotics. Studies of isolated human populations have shown that their microbiota is more diverse than that of industrialized groups and harbors genes conferring resistance to clinically relevant antibiotics (13–15). We wished to explore whether parallel changes would be found to have occurred in comparisons of wild-living primates to their captive-living relatives. Access to samples from wild baboons in Zambia and from captive baboons in the United States and to published metagenomic data sets from humans and baboons allowed us to test the hypothesis that human contact is correlated with substantial shifts in microbiota composition, function, and resistome profiles compared to “naive” baboon gut microbiota.

## RESULTS

### Comparison of the human and baboon gut microbiota.

**FIG 1:**
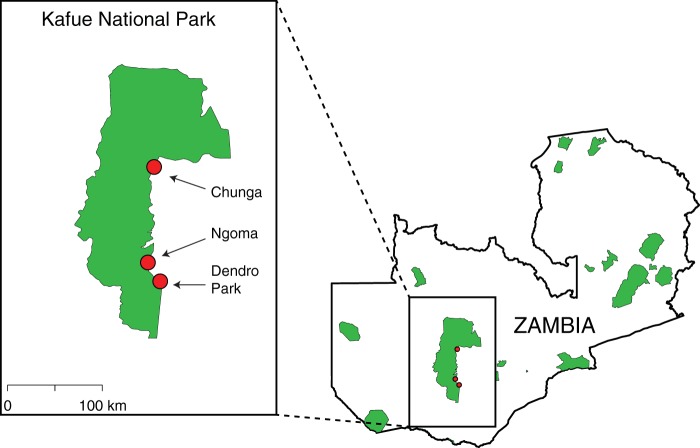
Location of the study sites in Kafue National Park, Zambia.

**FIG 2:**
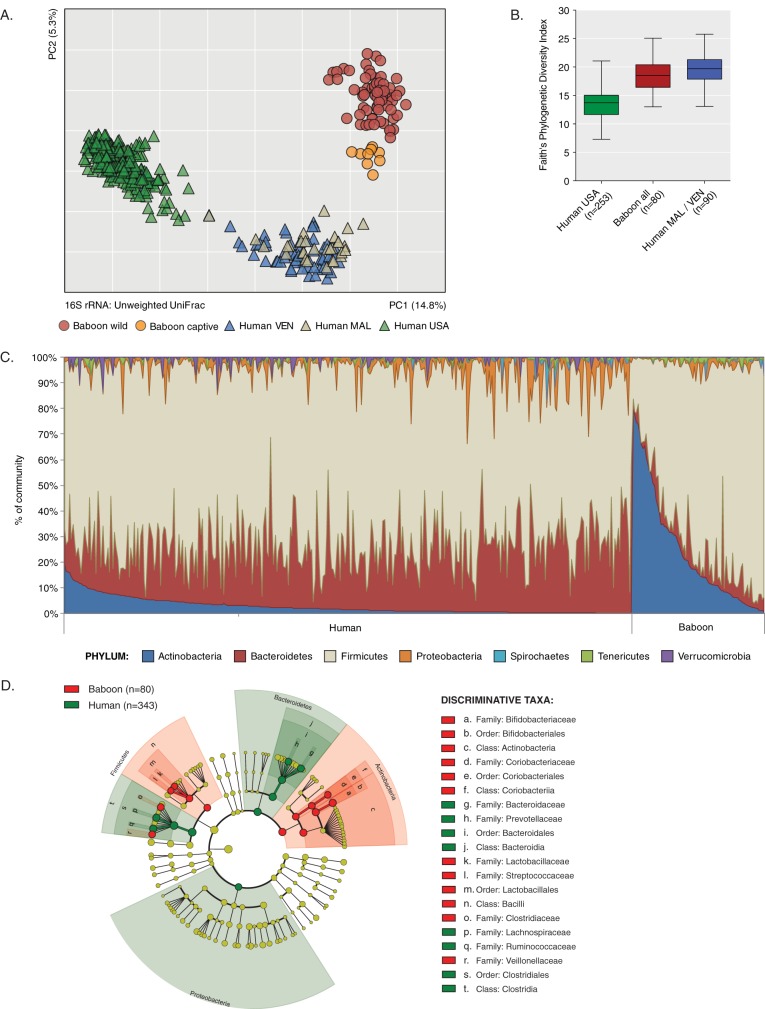
Taxonomic composition of the baboon and human gut microbiota. (A) Principal-coordinate analysis (PCoA) plot of 16S-based profiles from individual baboon and human fecal samples (United States [USA], Venezuela [VEN], Malawi [MAL]). (B) Alpha diversity (Faith’s phylogenetic diversity index) in baboons and humans. (C) Relative abundances of major bacterial phyla across individuals, sorted by decreasing abundance of *Actinobacteria*. (D) Phylogenetic tree of bacterial taxa identified in human and baboon 16S rRNA data sets. Clades significantly enriched (LEfSe; linear discriminant analysis [LDA] log score of >4.0, *P* = 0.05) in baboon (red nodes) and human (green nodes) communities are indicated. Yellow nodes denote clades not enriched in either group.

We performed sequencing of the 16S rRNA V4 region to survey the fecal microbiota of wild baboons (*n* = 71) from three localities in Zambia (Fig. 1) and from captive baboons (*n* = 9) from the Southwest National Primate Research Center (SNPRC; Texas, USA) (see Table S1 in the supplemental material). We analyzed our 16S data set along with data from a study of the microbiota of human adults living in urban areas in the United States (*n* = 253) and rural communities in Malawi (*n* = 30) and Venezuela (*n* = 60) (16). Principal-coordinate analysis (PCoA) of unweighted UniFrac distances indicates that the composition of the baboon gut microbiota is highly divergent from that of the two previously observed human microbiota clusters (urban versus rural) (Fig. 2A; analysis of similarity [ANOSIM] *R* = 0.91, *P* = 0.001). The phylogenetic diversity (Faith’s PD) of the baboon microbiota was higher than that seen with U.S. individuals (*t* test, *P* < 0.0001) and lower than that seen with the Malawi/Venezuela populations (*t* test, *P* = 0.0045) (Fig. 2B).

10.1128/mSystems.00016-18.5 TABLE S1  Metadata on the 80 baboons analyzed in the study. Age class was determined based on dental eruption patterns. Level of human contact is explained in Materials and Methods. Download TABLE S1, XLSX file, 0.03 MB. Copyright © 2018 Tsukayama et al. 2018 Tsukayama et al. This content is distributed under the terms of the Creative Commons Attribution 4.0 International license.

Like those of the human microbiota, baboon microbial communities display high interindividual variation in the relative abundances of bacterial phyla (Fig. 2C; see also Fig. S1A in the supplemental material) (16, 17). The *Firmicutes* phylum was the most abundant across all samples (Fig. 2C; see also Fig. S1A). However, at the ordinal level, linear discriminant analysis (LDA) performed using linear discriminant analysis effect size (LEfSe) (18) indicated that the baboon gut microbiota was enriched for *Lactobacillales* (families *Streptococcaceae* and *Lactobacillaceae*) (Fig. 2D), unlike those of humans, where members of the *Clostridiales* (families *Lachnospiraceae* and *Ruminococcaceae*) were enriched. Also, levels of members of the *Bacteroidetes* and the *Proteobacteria*, two bacterial phyla prominent in the healthy human gut, were greatly reduced in baboon communities. Actinobacterial species of the families *Bifidobacteriaceae* and *Coriobacteriaceae* were enriched in the wild baboon microbiota, reaching relative abundances as high as 80% in some individuals.

10.1128/mSystems.00016-18.1 FIG S1  (A) Distribution of relative abundances of the major bacterial phyla identified in human (*n* = 343), wild baboon (*n* = 71), and captive baboon (*n* = 9) gut communities, as determined by 16S rRNA sequencing. Boxplot whiskers indicate minimum and maximum values. *, *P* < 0.05 (nonparametric Student’s *t* test). (B and C) Alpha-diversity comparisons between wild (*n* = 71) and captive (*n* = 9) baboons at a rarefaction depth of 4,100 sequences. (B) Mean Faith’s PD index. (C) Number of observed OTUs. Download FIG S1, PDF file, 0.4 MB. Copyright © 2018 Tsukayama et al. 2018 Tsukayama et al. This content is distributed under the terms of the Creative Commons Attribution 4.0 International license.

**FIG 3:**
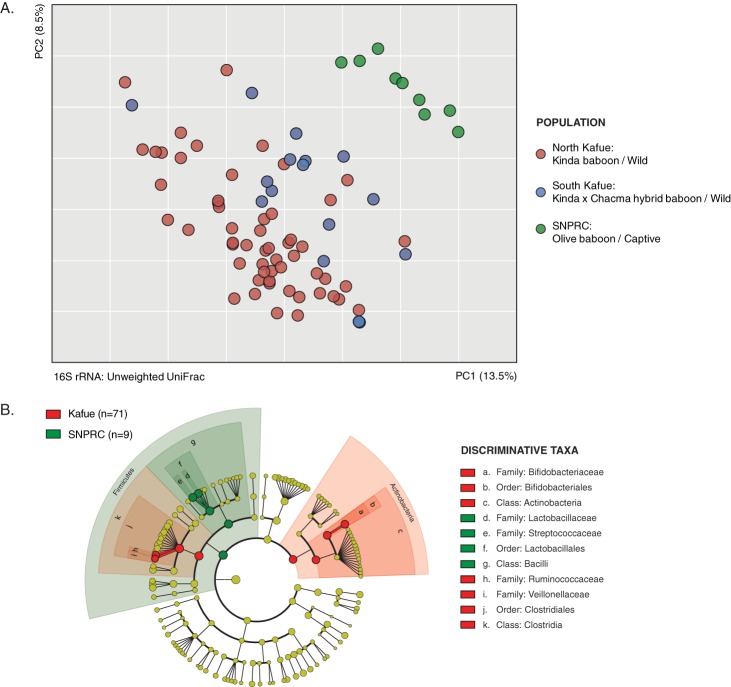
SNPRC baboons harbor a unique microbiota composition. (A) PCoA plot of Kafue (north, red circles, *n* = 55; south, blue circles, *n* = 16) and SNPRC (green circles, *n* = 9) baboon gut communities. (B) Phylogenetic tree of bacterial taxa (kingdom, phylum, class, order, family) identified in wild and captive baboon data sets. Clades significantly enriched (LDA log score of >4.0, *P* < 0.05) in all Kafue (red nodes) and SNPRC (green nodes) communities are indicated. Yellow nodes denote clades not enriched in either group.

Comparison of the fecal microbiota of wild Kinda baboon populations from Kafue National Park to that of captive olive baboons from SNPRC showed that the composition of their microbiota varies by site (ANOSIM *R* = 0.76, *P* = 0.001) (Fig. 3A), with lower species diversity in the wild group (Fig. S1B and C). At the 16S level, captive baboons form a discrete subpopulation that is more similar to humans from Malawi and Venezuela than to wild baboons (Fig. 2A), as indicated by pairwise comparison of mean Unifrac distances between groups (*t* value of −16.6, *P* < 0.005). The relative abundance of *Actinobacteria* species in captive baboons was the lowest among all sampled animals (Fig. S1A). The microbiota of the captive baboons was enriched in lactic acid bacteria of the order *Lactobacillales* (Fig. 3B). 16S rRNA profiles did not cluster significantly based on age class (ANOSIM *R* = 0.04, *P* = 0.273) or sex (*R* = 0.005, *P* = 0.397), but we observed significant clustering by social group membership (*R* = 0.52, *P* = 0.001) (Fig. S2C), as reported previously for wild baboon and chimpanzee populations (19, 20).

10.1128/mSystems.00016-18.2 FIG S2  Principal-coordinate plot of wild baboon microbiota and captive baboon microbiota from Kafue National Park and SNPRC. Samples are colored by (A) sex, (B) age class, and (C) social group. ANOSIM *R* values indicate the strength of sample grouping data by category. Download FIG S2, PDF file, 0.6 MB. Copyright © 2018 Tsukayama et al. 2018 Tsukayama et al. This content is distributed under the terms of the Creative Commons Attribution 4.0 International license.

### Functional selections and whole-metagenome surveys of AR genes suggest overlapping resistomes in humans and captive baboons.

We sought to identify AR genes in the baboon gut microbiota using two complementary methods: (i) performing functional metagenomic selections to discover genes that confer phenotypic resistance in Escherichia coli expression libraries and (ii) surveying whole metagenomes for AR genes present in curated databases.

**FIG 4:**
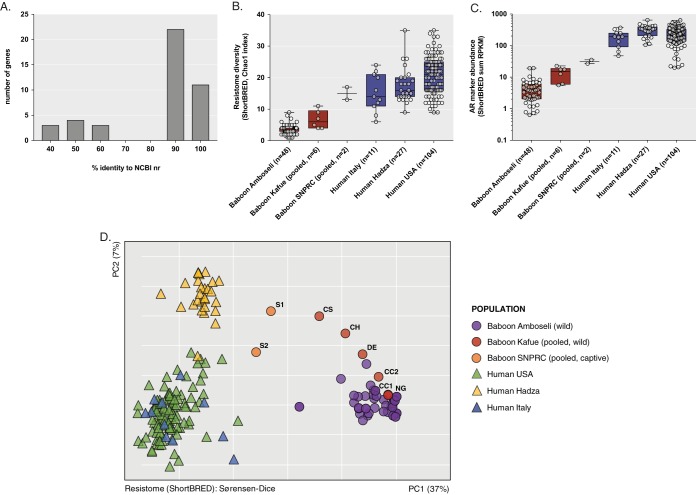
The antibiotic resistomes of baboons and comparison to human resistomes. (A) Percent identity of the 43 unique AR genes found in functional metagenomic selections of baboon libraries searched against the NCBI nr protein sequence database. (B) Resistome diversity per metagenome, based on markers generated from functional selections and the CARD resistance database. (C) Sum of all marker abundances per metagenome, expressed in RPKM. (D) PCoA plots of Sørensen-Dice similarity matrices from ShortBRED results. Kafue (wild) and SNPRC (captive) baboon metagenomes represent pooled libraries containing 18 and 6 individual baboons, respectively.

Due to the limited amount of fecal material available from individual animals from which to create expression libraries, we pooled metagenomic DNA in sets of three animals to create a total of eight pooled-DNA libraries. Pools corresponded to the six sampled baboon social groups: NG and DE for southern Kafue (low human contact); CC1, CC2, CH, and CS for northern Kafue (medium contact); and S1 and S2 for SNPRC (high contact) (Fig. S3; see also Table S1). Libraries were screened against 12 natural and synthetic antibiotics from six different classes (Table S2). Sequencing and assembly of resistance-conferring fragments using PARFuMs (21) resulted in 155 DNA contigs (mean length, 2.0 ± 1.3 kb) across all samples, of which 128 were unique (99% nucleotide identity clustering). No phenotypic resistance was observed for ciprofloxacin (a synthetic fluoroquinolone), meropenem (a late-generation carbapenem), tigecycline (a semisynthetic derivative of tetracycline), and colistin (a polymyxin) in any of the sampled baboon metagenomic libraries. Annotation of resistance contigs with Resfams (22) revealed that 49 (38%) contained AR genes that could be annotated with high confidence to a specific gene category and function (e.g., class A and C β-lactamases, chloramphenicol acetyltransferase, TetA efflux pump, TetM/W/O/S family of ribosomal protection factor, etc.) Ten of these predicted proteins were novel (<70% amino acid identity to any protein in NCBI nr) (Fig. 4A): six chloramphenicol acetyltransferases and four 16S rRNA methyltransferases. Assembly of regions flanking AR genes enabled the annotation of mobile genetic elements (MGEs; e.g., integrases, transposases, phage recombinases) in 15 (11.7%) contigs from CH and CS (northern Kafue, medium contact), and S1 and S2 (SNPRC, high contact) libraries. Four of these MGEs were syntenic with class A β-lactamases and the TetO ribosomal protection protein. No putative MGEs were identified in resistance contigs from southern Kafue (low contact) baboon metagenomes. Table S3 shows the full set of resistance contigs and annotations.

10.1128/mSystems.00016-18.3 FIG S3  16S-based taxonomic profiles of individual samples pooled for functional metagenomic analysis and whole-metagenome sequencing. (A) Relative abundances of major bacterial phyla. Columns represent individual baboon samples, grouped by social group. (B) PCoA plot based on unweighted UniFrac distances. White circles indicate baboon samples that were not used for metagenomic analyses. Download FIG S3, PDF file, 0.5 MB. Copyright © 2018 Tsukayama et al. 2018 Tsukayama et al. This content is distributed under the terms of the Creative Commons Attribution 4.0 International license.

10.1128/mSystems.00016-18.6 TABLE S2  Antibiotics used in functional metagenomic selections for AR genes. Screening concentrations were defined as the minimum concentrations shown to inhibit the growth of control E. coli MegaX DH10B in our control plates. Download TABLE S2, XLSX file, 0.01 MB. Copyright © 2018 Tsukayama et al. 2018 Tsukayama et al. This content is distributed under the terms of the Creative Commons Attribution 4.0 International license.

10.1128/mSystems.00016-18.7 TABLE S3  Summary of 128 unique resistant-conferring contigs found in pooled baboon fecal libraries. Contigs were assembled with PARFuMS and annotated with Resfams v1.2. Known antibiotic resistance genes are highlighted in red. Elements associated with mobile genetic elements are highlighted in green. Download TABLE S3, XLSX file, 0.1 MB. Copyright © 2018 Tsukayama et al. 2018 Tsukayama et al. This content is distributed under the terms of the Creative Commons Attribution 4.0 International license.

We performed shotgun metagenomic sequencing on the same eight pooled samples to further characterize their resistomes and analyze them in the context of published metagenomes from baboon and human cohorts. ShortBRED (23) was used to conduct a metagenome-wide survey of AR gene composition and abundance. First, we created unique protein markers from a combination of 43 unique annotated AR proteins identified by functional metagenomics in baboon expression libraries and the 2,165 reference AR protein sequences in the Comprehensive Antibiotic Resistance Database (CARD) (24); this extended our resistome analysis to antibiotics that target Gram-positive bacteria (e.g., vancomycin, macrolides, lincosamides, streptogramins) and to any other known AR genes that are not detectable in functional selections in our E. coli host. We then measured the abundance of these markers across the shotgun-sequenced metagenomes of (i) our eight pooled baboon fecal communities used in functional selections, (ii) 48 wild yellow baboons from Amboseli National Park in Kenya (20), (iii) 104 U.S. human metagenomes from the first-phase Human Microbiome Project (17), and (iv) 38 metagenomes from a study comparing Hadza hunter-gatherer groups in northwestern Tanzania (*n* = 27) to healthy Italian adults (*n* = 11) (25). See Materials and Methods for details of the ShortBRED analysis and criteria for AR marker selection.

Hits for 114 AR markers were found across all sampled metagenomes. Human samples had ~5×-greater AR gene marker richness than baboon samples (Chao1 indices, 19.8 ± 0.5 versus 4.3 ± 0.4, *P* < 0.0001) (Fig. 4B; see also Fig. S4A), and these markers were ~37× more abundant in humans (246.5 ± 10.8 versus 6.5 ± 0.9 cumulative ShortBRED reads per kilobase per million [RPKM], *P* < 0.0001) (Fig. 4C; see also Fig. S4B). Genes conferring resistance to aminoglycoside, MLS, β-lactam, and tetracycline antibiotics were abundant in samples from the United States and Italy. Although the population was less extensively exposed to clinical antibiotic use, the Hadza hunter-gatherer microbiota also showed a high abundance of AR gene markers, as originally reported (25). Among baboons, the most common and abundant markers were an OXA-type (class D) β-lactamase and a novel chloramphenicol acetyltransferase. Notably, the two SNPRC (captive, high contact) metagenomes had the highest number and highest abundance of ShortBRED hits, followed by the Chunga School pooled metagenome (wild, medium contact). These contained AR markers that were not usually present in wild baboon metagenomes and were common and abundant in human metagenomes (e.g., CfxA6 β-lactamase and TetW/TetO ribosome protection factors). The full list of ShortBRED markers and their abundances is shown in Table S4.

10.1128/mSystems.00016-18.4 FIG S4  Whole-metagenome surveys of microbiome composition and function. (A to C) Comparison of resistomes (A and B) and microbiota genus diversity (C) between human and baboon metagenomes. (D and E) PCoA plots of (D) microbiota composition profiles, calculated from the presence and absence of MetaPhlan2 species markers and (E) microbial functional profiles, based on the presence of UniRef90 gene families determined using HUMAnN2. Download FIG S4, PDF file, 0.8 MB. Copyright © 2018 Tsukayama et al. 2018 Tsukayama et al. This content is distributed under the terms of the Creative Commons Attribution 4.0 International license.

10.1128/mSystems.00016-18.8 TABLE S4  Normalized abundances of 118 AR gene markers across baboon (*n* = 56) and human (*n* = 140) metagenomes, as determined by ShortBRED, sorted by antibiotic class. Download TABLE S4, XLSX file, 0.1 MB. Copyright © 2018 Tsukayama et al. 2018 Tsukayama et al. This content is distributed under the terms of the Creative Commons Attribution 4.0 International license.

PCoA ordination of Sørensen-Dice index (presence/absence) matrices based on ShortBRED outputs indicated that baboon and human resistomes are, in general, different from each other (ANOSIM *R* = 0.96, *P* = 0.001; Fig. 4D). However, resistomes from captive baboons (S1 and S2) were more similar in their composition to Hadza hunter-gatherer resistomes than to resistomes from other baboons (mean Sørensen-Dice index, 0.49 ± 0.02 versus 0.69 ± 0.03).

To leverage our much larger 16S data set, we used PICRUSt (26) to infer metagenomes from taxonomic profiles and performed LDA of enriched KEGG ortholog (KO) categories in comparisons between predicted captive and wild baboon metagenomes (Table S5). Consistent with our shotgun and functional resistome analysis, we find that the second most discriminatory feature for captive baboons was KO K12555, which encodes penicillin-binding protein 2A, associated with resistance to β-lactam antibiotics. Grouping predicted metagenome features at the pathway level, we identified nine differentially enriched features for captive baboons. The fifth most discriminative pathway was “Penicillin and cephalosporin biosynthesis” (Table S6).

10.1128/mSystems.00016-18.9 TABLE S5  KEGG orthologs (KOs) differentially enriched (LDA score of >3.5) in wild and captive baboon PICRUSt-predicted metagenomes. Download TABLE S5, XLSX file, 0.01 MB. Copyright © 2018 Tsukayama et al. 2018 Tsukayama et al. This content is distributed under the terms of the Creative Commons Attribution 4.0 International license.

10.1128/mSystems.00016-18.10 TABLE S6  KEGG pathways differentially enriched (LDA score of >2.0) in wild and captive baboon PICRUSt-predicted metagenomes. Download TABLE S6, XLSX file, 0.01 MB. Copyright © 2018 Tsukayama et al. 2018 Tsukayama et al. This content is distributed under the terms of the Creative Commons Attribution 4.0 International license.

The similarity between the resistomes of captive baboons and humans prompted us to extend the metagenomic analysis from antibiotic resistance functions to microbiota composition and metabolic gene pathways. We used MetaPhlAn2 (27) to assess the microbial species composition of these metagenomes and observed that (i) human and baboon microbiota were markedly different in their composition (ANOSIM *R* = 0.75, *P* = 0.01; see also Fig. S4D); (ii) Hadza hunter-gatherers formed a separate cluster from HMP/Italy metagenomes (ANOSIM *R* = 0.88, *P* = 0.01); (iii) bacteria of the orders *Bifidobacteriales* and *Lactobacillales* were enriched in baboon samples, while the order *Bacteroidales* was enriched in humans (LEfSe, LDA log score >4.5, *P* = 0.05); and (iv) captive baboons were more similar in their microbiota composition to Hadza humans than to other baboons (mean Sørensen-Dice index, 0.48 ± 0.01 versus 0.54 ± 0.03; see also Fig. S4D). We used HUMAnN2 (28) to perform functional profiling of metagenomes by mapping translated DNA reads to the UniRef90 and MetaCyc databases (29) and determined the presence of cataloged gene families. Comparison of Sørensen-Dice matrices from HUMAnN2 outputs showed that (i) functional profiles of baboon and human metagenomes also clustered apart strongly (ANOSIM *R* = 0.73, *P* = 0.001) and (ii) baboons from this study (Kafue, SNPRC) had HUMAnN2 profiles that were more similar to those of Hadza humans than to those of Amboseli baboons (mean Sørensen-Dice index, 0.53 ± 0.02 versus 0.67 ± 0.02; see also Fig. S4E). We used LefSe (18) to identify microbial metabolic pathways that were enriched in baboon microbiomes compared to humans. Among the 20 most discriminant pathways (LDA score of >3.5, *P* < 0.05), baboons were enriched in the pathway of pyruvate fermentation to acetate and lactate (Table S6).

## DISCUSSION

### Baboon microbiota architectures across different habitats and lifeways.

Vertebrate hosts and their microbes have coexisted for millions of years, resulting in the adaptation of gut commensal populations to diverse host lifeways across the animal kingdom (30–32). This coadaptation is evident in humans, where marked differences in microbial community composition occur across a spectrum of diets, cultures, and geography (16, 33, 34). Comparison to published human data sets enabled the analysis of baboon 16S rRNA results in the context of modern humans. Access to wild baboon populations from Zambia and captive animals from SNPRC allowed us to study the variation of the baboon microbiota across different habitats, lifeways, diets, and levels of human contact. The finding that the baboon gut microbiota is distinct from that of humans is in line with other studies in primate populations (17). However, within the baboon cluster, captive animals formed a separate group represented by a different microbial composition, possibly reflecting differences in habitat and lifeways compared to wild baboons from Kafue. We observed lower alpha-diversity in the microbiota of baboons compared to humans (see Fig. S4C in the supplemental material) and in wild baboons compared to captive ones (Fig. S1), in both 16S and MetaPhlan2 data sets. This is in contrast to recent reports describing loss of microbial species diversity in captive primates compared to wild ones (19) and in Western human populations compared to non-Western groups (14–16). However, captivity is not always associated with reduced diversity in mammals (35), and a more diverse microbiota may not necessarily correlate with increased fitness for the host in a given environment.

The high relative abundance of bifidobacteria in baboon populations was an unexpected finding (Fig. S1) that was, however, observed in two independent data sets (16S and MetaPhlAn2) and corroborated in a recent report (36). This group comprises some of the most abundant species in the infant human gut, which decrease substantially as the microbiota reaches a mature configuration in the first 3 years of life (16, 37), consistent with its proposed role in the fermentation of milk oligosaccharides (38, 39). We observed high relative abundances of bifidobacteria despite most sampled baboons being adults or juveniles (only three baboons were infants, and those did not harbor significantly different gut communities) (Fig. S2). This finding suggests that bifidobacteria may play other ecological roles in the baboon microbiota beyond the digestion of milk oligosaccharides during early life stages, such as protection from enteric pathogens via production of fermentation end products (40). Indeed, using HUMAnN2, we saw an enrichment of enzymes related to bacterial pyruvate fermentation to acetate and lactate, a feature commonly associated with bifidobacteria and lactic acid bacteria (41). Levels of *Actinobacteria* in captive baboons were much lower than in wild groups, suggesting that conditions that select for the high abundance of bifidobacteria in the microbiota of wild animals are absent in captivity.

### Resistome profiles in human and baboon populations.

Previous surveys of human, animal, and environmental resistomes supported the view that antibiotic resistance is a ubiquitous feature of microbial communities even in the most remote locations (5, 14, 42). Genes encoding resistance are presumably maintained in the absence of inhibitory concentrations of antibiotics, perhaps by playing alternative roles in these ecosystems (3, 9, 43).

We hypothesized that exposure to humans and their activities results in shifts in microbiota composition and function and in an expansion of the baboon gut resistome. We used culture-independent metagenomic methods and bioinformatic tools to survey the resistomes of baboon populations from different habitats and at various levels of human contact. While contact may be partly responsible for the differences observed across baboon metagenomes, it is possible that differences in habitat, diet, host species, and social interactions may also contribute to the observed differences (20, 36, 44).

We acknowledge that there is a bias in AR gene databases toward genes found in human pathogens. This is one of the reasons why we performed functional selections of eight pooled baboon metagenomes. By surveying functional resistance phenotypes, these selections can identify novel, unannotated genes conferring resistance. Using this approach, we observed phenotypic chloramphenicol resistance in metagenomes from baboons with low human contact (southern Kafue), whereas the metagenomes of animals in areas of medium and high human contact showed resistance to seven other antibiotics, including newer-generation β-lactams and cephalosporins. Previously identified AR genes were annotated in only ~40% of resistance contigs, and 10 previously unknown resistance proteins were identified in our experiments (Fig. 4A), highlighting the novelty of the baboon resistomes and the need to further characterize nonhuman and environmental resistomes.

Metagenome-wide surveys of AR genes identified in functional selections and the CARD resistance gene database allowed the comparison of Kafue baboon metagenomes to data sets from previous baboon and human studies and identified resistance genes not captured in functional selections. This approach identified AR genes in all sampled baboon gut microbiomes. We also analyzed the metagenomes of 48 yellow baboons from Amboseli National Park in Kenya and found fewer AR genes than were present in the metagenomes of baboons in Kafue National Park. However, several Amboseli baboons contained class A, B, and D β-lactamases commonly associated with plasmids and integrons found in human pathogens (24). Like the baboons in Kafue National Park, Amboseli baboons live in a protected area away from large human settlements. However, these animals have come into contact with researchers (20), tourists, and local pastoralist groups for decades (45), creating multiple opportunities for the transfer of AR genes between human and baboon gut microbiota.

Our resistome surveys also showed that microbiota from wild baboons of the Chunga group (CS, medium contact) and captive baboons from SNPRC (S1 and S2, high contact) had greater diversity and abundance of AR genes than microbiota from baboons of low-contact groups. Many of these genes are also found in human gut metagenomes and human bacterial pathogens, and are commonly associated with mobile genetic elements (Tables S5 and S6) (24). Overall, resistome, taxonomic composition, and functional profiles of captive baboon microbiomes were more similar to those of Hadza hunter-gatherers than to those of wild baboons (Fig. 4D; see also Fig. S4). Captive baboon microbiota contained genes encoding class A and D β-lactamases, aminoglycoside nucleotidyltransferases, tetracycline efflux pumps, and ribosome protection factors that were also present in sampled human gut metagenomes in our analyses. It is plausible that a subset of these AR genes and bacterial taxa were not native to baboon gut microbiota but were rather exchanged with caretakers at SNPRC and sympatric humans at Chunga in recent times. Our findings support the hypothesis that sharing habitat with human populations—and the lifeways and diet changes that result from such sharing—may lead to a “humanization” of the primate gut microbiota (44) and its antibiotic resistome, although the consequences of these population shifts for overall host health remain unknown.

## MATERIALS AND METHODS

### Sample collection and study design.

We obtained fecal samples from wild baboon populations from Kafue National Park in Zambia as part of a field-based study of the Kinda baboon (Papio kindae) and the grayfoot chacma baboon (Papio ursinus griseipes). The genus *Papio* includes six clearly distinguishable, phylogenetically distinct major taxa, which nevertheless are interfertile and can and do interbreed in the wild (46). Under some species definitions (which we prefer), they are considered separate and yet closely related species; others prefer to regard them as subspecies of a single species. Kinda and chacma baboon populations come into contact in the Kafue National Park, and interbreeding occurs between them, producing a hybrid zone in which individuals of mixed appearance and parentage are found (47). We collected samples from 55 Kinda baboons from three social groups that live in proximity to humans near the Kafue National Park (North) Headquarters at Chunga (15°2′S, 26° 0′E) (Chunga School, Chunga HQ, and Chunga College groups) and from 16 hybrids living near the National Park (South) Headquarters at Ngoma (15°58′S, 25°56′E) (*n* = 12) and near the Dendro safari camp (16°9′S, 26°4′E) (*n* = 4) located approximately 100 km south of Chunga (Fig. 1). Samples from Chunga groups (northern Kafue) were collected in May and June 2011. Samples from Ngoma and Dendro groups (southern Kafue) were obtained in May and June 2012. Diet in all baboons was varied and consisted mostly of fruits, pods, seeds, leaves, corms, and, rarely, animal protein. The Chunga School and HQ groups had overlapping ranges and supplemented their diet considerably by feeding on discarded human foods at garbage dumps (J. E. Phillips-Conroy, C. J. Jolly, and J. Rogers, personal observations). Baboons were temporarily captured and tranquilized to allow specimen collection and to determine their sex and age based on dental eruption patterns. We also obtained samples from nine captive olive baboons (Papio anubis) from SNPRC in San Antonio, TX, along with information on their age, diet, and medical history. Captive baboons were fed a commercial chow preparation (15% protein, 4% fat, 10% crude fiber) and had received one or more courses of antibiotics (penicillin-G, amoxicillin, ceftriaxone, cephalexin, cefpodoxime, cefazolin, metronidazole, orbifloxacin, enrofloxacin) throughout their lifetime, as described in individual medical records. We define the baboons sampled from Ngoma and Dendro (southern Kafue) as “low human contact” because they have infrequent contact with only a few humans. We define the baboons sampled from Chunga (northern Kafue) as “medium human contact” because their range encompasses a village of several hundred humans and they regularly feed on discarded human foods. We define the baboons sampled from SPNRC as “high human contact” because these baboons were born and bred in captivity with constant human control of their diets and environment. Fecal samples were collected into sterile containers and stored in liquid nitrogen until shipment to Washington University in St. Louis, where samples were kept at −80°C until processing. Baboon metadata are summarized in Table S1 in the supplemental material. Baboon sample collection was conducted with the permission of the Zambian Wildlife Authority and in compliance with institutional animal care and use committee requirements at Washington University, New York University, and Baylor College of Medicine.

### DNA extraction and 16S rRNA phylogenetic analysis.

Metagenomic DNA was extracted from 250 mg of each specimen using a standard phenol-chloroform bead-beating protocol (48) and eluted in Qiagen EB buffer. The 16S rRNA V4 region (positions 515 to 806) was amplified using bar-coded primers and PCR protocols described previously (49). Each reaction mixture contained 12.5 µl of Hot-Start *Taq* DNA polymerase mix (Takara-Clontech), 1 µl of forward and reverse primers (10 µM), 1.0 µl of genomic DNA (1 ng/µl), and 12 µl of nuclease-free water. Bar-coded amplicons were pooled and sequenced on an Illumina MiSeq instrument with 2 × 250-bp paired-end reads.

Bar-coded Illumina reads were demultiplexed in QIIME v.1.8 (50). Paired reads were quality filtered (*split_libraries_fastq.py -q0 –r 500 –p 0 –n 500*) and merged using USEARCH v7 (51). Operational taxonomic units (OTUs) were generated *de novo* to uncover novel taxa and were clustered at 97% sequence identity from all merged and filtered reads with the UPARSE pipeline (52). Representative sequences from each OTU were assigned taxonomy with UCLUST against the Greengenes database (version 13_8, 97% clusters), aligned, and used to create a phylogenetic tree in QIIME. The pipeline generated 1,109 OTUs across the 80 baboon samples. Samples were rarefied to 4,100 sequences per sample for community diversity analyses.

We compared our baboon 16S data to the GlobalGut human 16S data set generated from individuals in urban centers in the United States and rural communities in Malawi and Venezuela (16) (MG-RAST accession number: qiime:850). We excluded samples from infants younger than 3 years old because of the highly variable nature of the gut microbiota during postnatal development (16, 33). The remaining 343 samples (United States, 253; Venezuela, 60; Malawi, 30) were rarefied to 50,000 reads each. Baboon reads were trimmed to 101 bp and combined with the GlobalGut reads. *De novo* OTU tables were generated in QIIME and rarefied to 13,000 sequences per sample. Unweighted UniFrac distances (53) were calculated and used for principal-coordinate analysis. As an internal control, we analyzed 10 human samples from the United States (*n* = 4) and Peru (*n* = 6) from a previous study by our group (10) to verify that the observed differences between baboons and humans were not due to artifacts from cross-comparison with published 16S data sets (data not shown).

The analysis of similarity (ANOSIM) test was performed in QIIME to assess clustering of samples by host species, sex, age class, social group, and captivity status. Mean UniFrac distances between sampled groups were calculated to compare levels of microbiota similarity between cohorts. LEfSe (18) was used to identify overrepresented taxa in sampling groups (e.g., Kafue versus SNPRC baboons). It runs the nonparametric Kruskal-Wallis sum-rank test to detect features with significant (*P* < 0.05) differential abundance for the class of interest, followed by the Wilcoxon rank sum test to detect biological significance (*P* < 0.05) and linear discriminant analysis (LDA) to estimate the effect size of each differentially abundant taxon.

### Functional metagenomic selections.

Due to the limited amount of metagenomic DNA available from individual samples, we pooled samples from three baboons with similar 16S profiles from each of the six groups at Kafue and SNPRC (Fig. S3; see also Table S1). Two pools were created from the Chunga College group because it contained samples with high levels of *Actinobacteria*, a feature not typically observed in adult human gut metagenomes. Pool CC1 included three specimens in which the relative abundance of *Actinobacteria* was greater than 60%, while pool CC2 included samples with 20% to 40% relative abundance. Two pools (S1 and S2) were created from the SNPRC animals to increase the representation of captive baboon metagenomes in our analyses. Functional metagenomic libraries were prepared and sequenced as described in reference 54. Briefly, small-insertion (3 to 6 kb) expression libraries (0.4 to 3.1 GB, equivalent to ~80 to 700 E. coli K-12 genomes) were created from 20 µg of pooled metagenomic DNA in vector pZE21 in E. coli MegaX DH10B electrocompetent cells (21). Libraries were screened on Mueller-Hinton agar plates containing 12 natural and synthetic antibiotics from six different classes at concentrations previously determined to inhibit the growth of nontransformed MegaX cells (Table S2). Resistant colonies were pooled and subjected to PCR with vector-specific primers, bar-coded, and sequenced on an Illumina HiSeq 2000 instrument (2 × 101-bp reads). Reads were filtered, demultiplexed, and assembled into contigs with PARFuMS (21). Assembled contigs smaller than 500 bp were removed from further analysis. Predicted ORFs were annotated with Resfams v1.2 (22). ORFs that could be classified with high confidence as AR genes specific to the screened antibiotic class were clustered at 99% identity and used for ShortBRED marker creation. These sequences were compared to NCBI nr (accessed December 2017) with blastp to identify the top local hits. The global percent identity was calculated as the number of matches over the length of the shorter sequence. Matches with the highest alignment score and lowest E value were used for analysis.

### Metagenome-wide measurement of AR gene abundance.

Metagenomic DNA (500 ng) from the same eight baboon pools used for functional metagenomic selections was sheared to ~450 bp, bar-coded, and sequenced on an Illumina HiSeq 2500 instrument with 2 × 150-bp paired reads. Reads were demultiplexed with no mismatches and trimmed with Trimmomatic v0.36 (55) to remove Illumina adapters and low-quality bases using the following parameters: *trimmomatic-0.36.jar PE-phred33 ILLUMINACLIP: TruSeq3-PE.fa:2:30:10:1:TRUE LEADING:10 TRAILING:10 SLIDINGWINDOW:4:15 MINLEN:60*. Human and baboon sequences were removed with DeconSeq (56) by mapping to the human reference genome (GRCh38) and a published baboon genome (Papio anubis, *GCA_000264685.2 Panu_3.0*), resulting in 10,141,457 ± 1,200,437 cleaned reads per sample.

To measure the relative abundance of resistance genes, a set of 923 unique markers was generated with ShortBRED (23) from 2,208 antibiotic resistance protein sequences using *shortbred_identify.py* with a cluster identity of 95% and Uniref90 as a reference database. The protein sequences used for identification of marker families included 43 AR protein sequences identified via functional metagenomic selections in baboon samples and 2,165 AR protein sequences from the CARD database (24). The marker list was manually curated to reduce the rate of false positives in our surveys. Entries were removed if they were not associated with resistance phenotypes or had a low risk of transmission across environments, based on the criteria described by Martínez et al. (57). These included (i) genes that confer resistance via overexpression of resistant target alleles (e.g., resistance to antifolate drugs via mutated dihydropteroate synthase [DHPS] and dihydrofolate reductase [DHFR]); (ii) genes associated with global gene regulators, two-component system proteins, and signaling mediators; (iii) genes encoding efflux pumps that confer resistance to multiple antibiotics (those known to confer resistance to single antibiotic classes, such as the TetA family, were retained in the analysis); and (iv) genes associated with modifications of cell wall charge (e.g., those conferring resistance to polymyxins and defensins). The final marker list consisted of 687 unique sequences. In order to measure the abundance of these markers, *shortbred_quantify.py* script was used with our eight pooled baboon metagenomes and published metagenomes from yellow baboons (Papio cynocephalus) in Amboseli National Park in Kenya (*n* = 48) (20), from healthy adult volunteers from cities in the United States (*n* = 102) (17), and from a recent study that compared the microbiota of Hadza hunter-gatherers in Tanzania (*n* = 27) to the microbiota of Italian volunteers (*n* = 11) (25). Relative abundance tables were filtered for markers with RPKM values of <0.1, resulting in 114 AR markers with positive hits (Table S4). Output tables were converted to the BIOM format, and QIIME v1.8 was used to calculate beta diversity (*-m binary_sorensen_dice*)and to run ANOSIM tests and principal-coordinate analyses.

The use of pooled baboon metagenomes and their comparison to individual human and baboon samples could potentially result in bias in measuring marker abundance and low-frequency signals. We addressed this by (i) filtering low-abundance hits (RPKM < 0.1) and using a binary (presence/absence) metric (Sørensen-Dice index) as opposed to a quantitative metric (e.g., Bray-Curtis) and (ii) creating 15 *in silico* pooled triplets from Amboseli, HMP, and Hadza metagenomes, rarefied to one-third of the reads, and ran these with individual samples in metagenomic surveys. Abundance tables and principal-coordinate analyses showed that, using the same analysis parameters, detection levels and resistome profiles of pooled samples were similar to those of individual samples (Table S4).

### Prediction of metagenomes from 16S rRNA profiles.

We used the Galaxy implementation of PICRUSt v1.1.1 (http://galaxy.morganlangille.com/) to infer the metagenome composition based on 16S rRNA profiles. Closed-reference OTU tables were created in QIIME v1.8 using the GreenGenes 13.5 reference database. We performed normalization of 16S copy numbers, followed by metagenome prediction and grouping into L3 KEGG categories. LEfSe was used to identify KO entries of pathways that were differentially enriched in captive and wild baboon metagenomes.

### Taxonomic composition and functional profiling of baboon and human metagenomes.

MetaPhlAn2 (27) was used to assess the microbial composition of baboon and human metagenomes and to expand our analysis beyond 16S analysis to species-level resolution and nonbacterial microbial taxa. To infer the functional profiles of baboon and human metagenomes, HUMAnN2 (28) analysis was performed by calculating relative abundances of annotated microbial gene families and pathways in the UniRef90 (29) and MetaCyc (58) databases. We normalized (*humann2_renorm_table.py*) merged individual “pathway abundance” and “gene family” output files (*humann2_join_tables.py*), converted them to BIOM format, and filtered hits with relative abundance corresponding to RPKM values of <0.1. QIIME 1.8 was used to calculate Sørensen-Dice distances (*beta_diversity.py –m binary_sorensen_dice*), to run ANOSIM tests (*compare_categories.py*), and to perform principal-coordinate analysis (*principal_coordinates.py*). LefSe (18) was used to identify biomarker taxa, AR genes, and metabolic pathways from humans and baboons in metagenomic data sets.

### Accession number(s).

Raw sequence reads generated for this study have been deposited with accession numbers PRJNA430956 and PRJNA454115 in the NCBI BioProject database.
