# Supplementary figures and images for: Characterization of Wild and Captive Baboon Gut Microbiota and Their Antibiotic Resistomes
Source: mSystems. 2018 Jun 26;3(3):e00016-18. doi: 10.1128/mSystems.00016-18 (PMC6020475; doi:10.1128/mSystems.00016-18)

Fig S1

A.

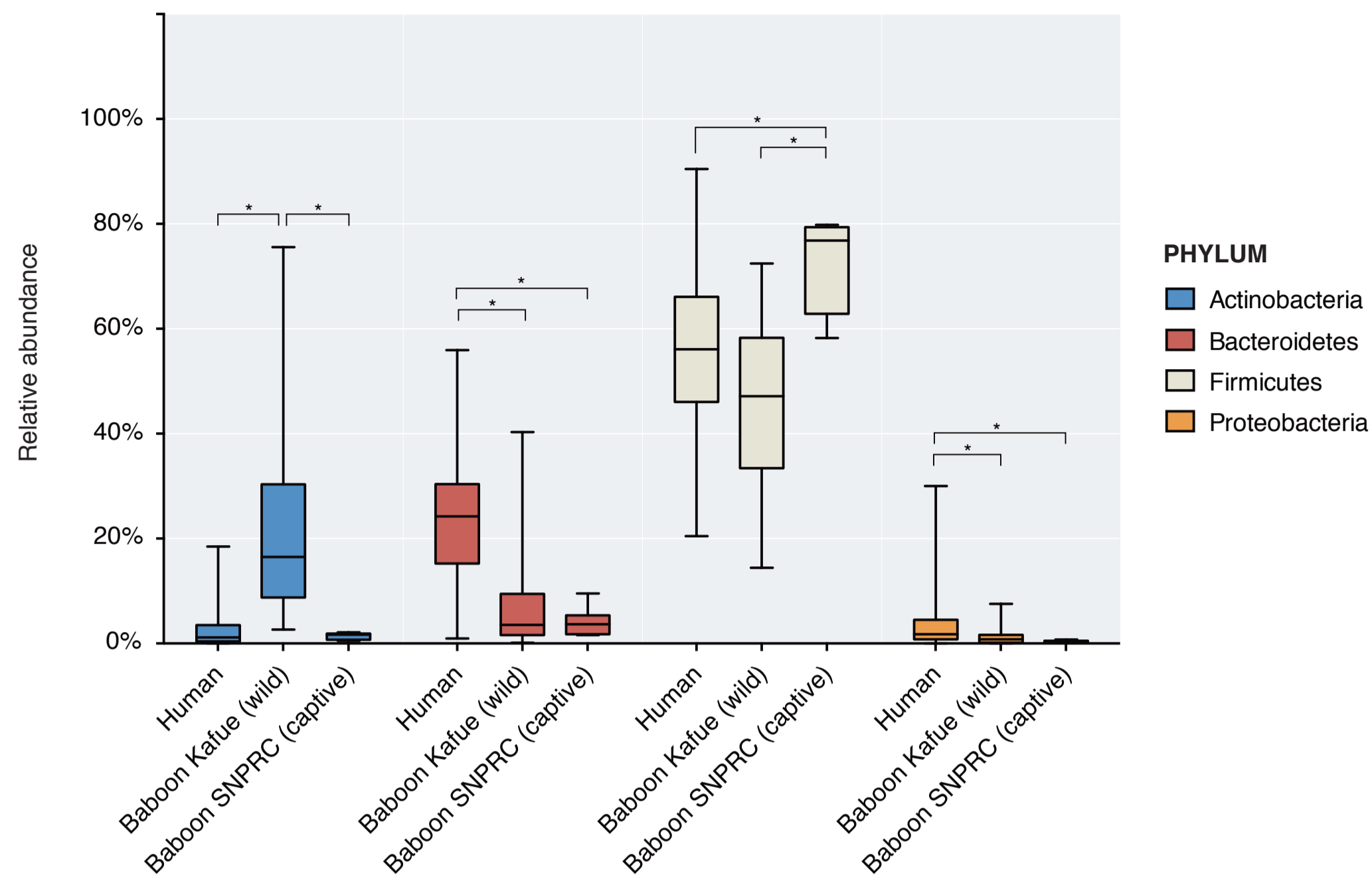

B.

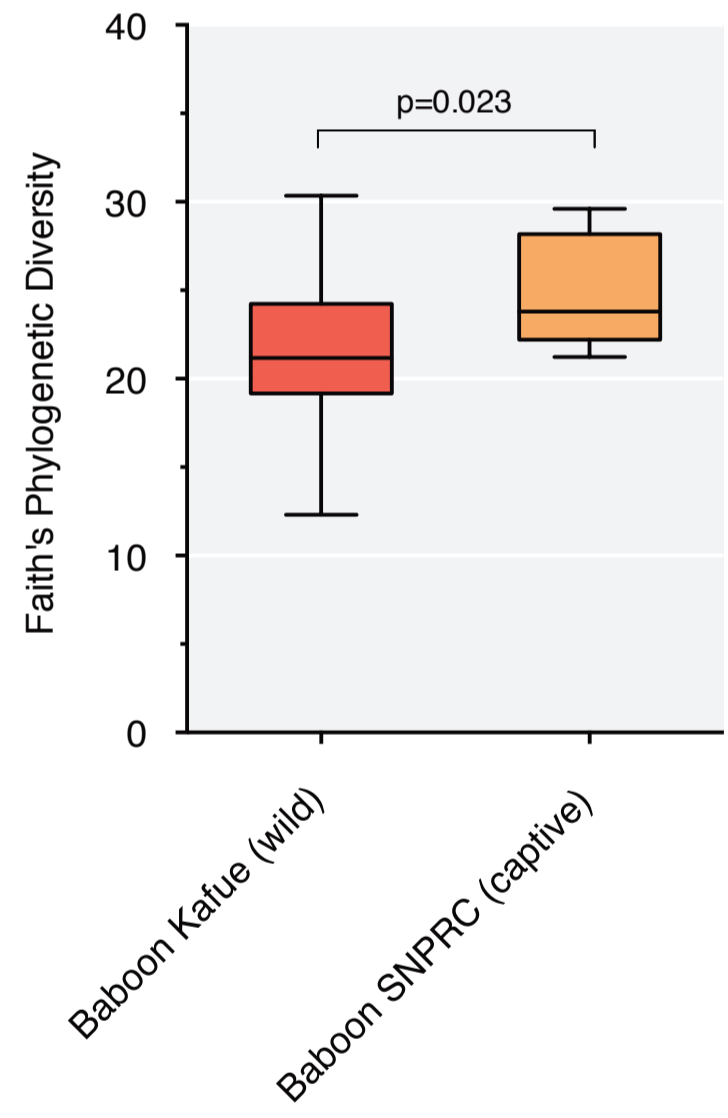

C.

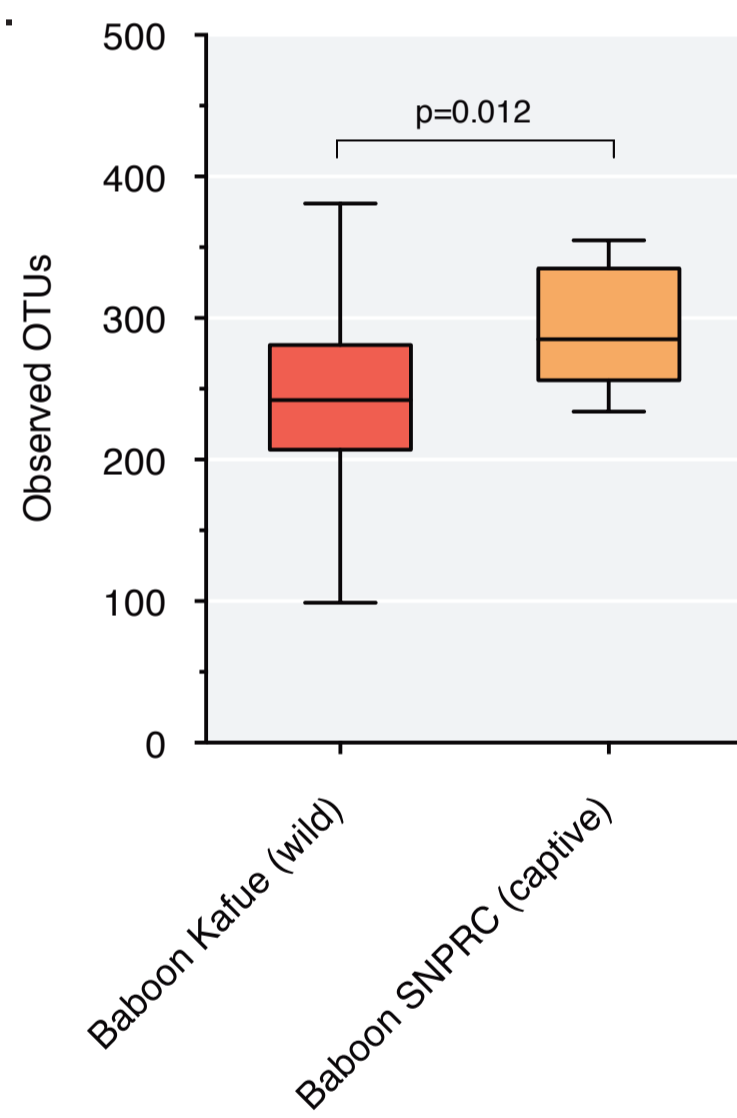

Supplement: FIG S1 [file sys003182241sf1.pdf]

Fig S2

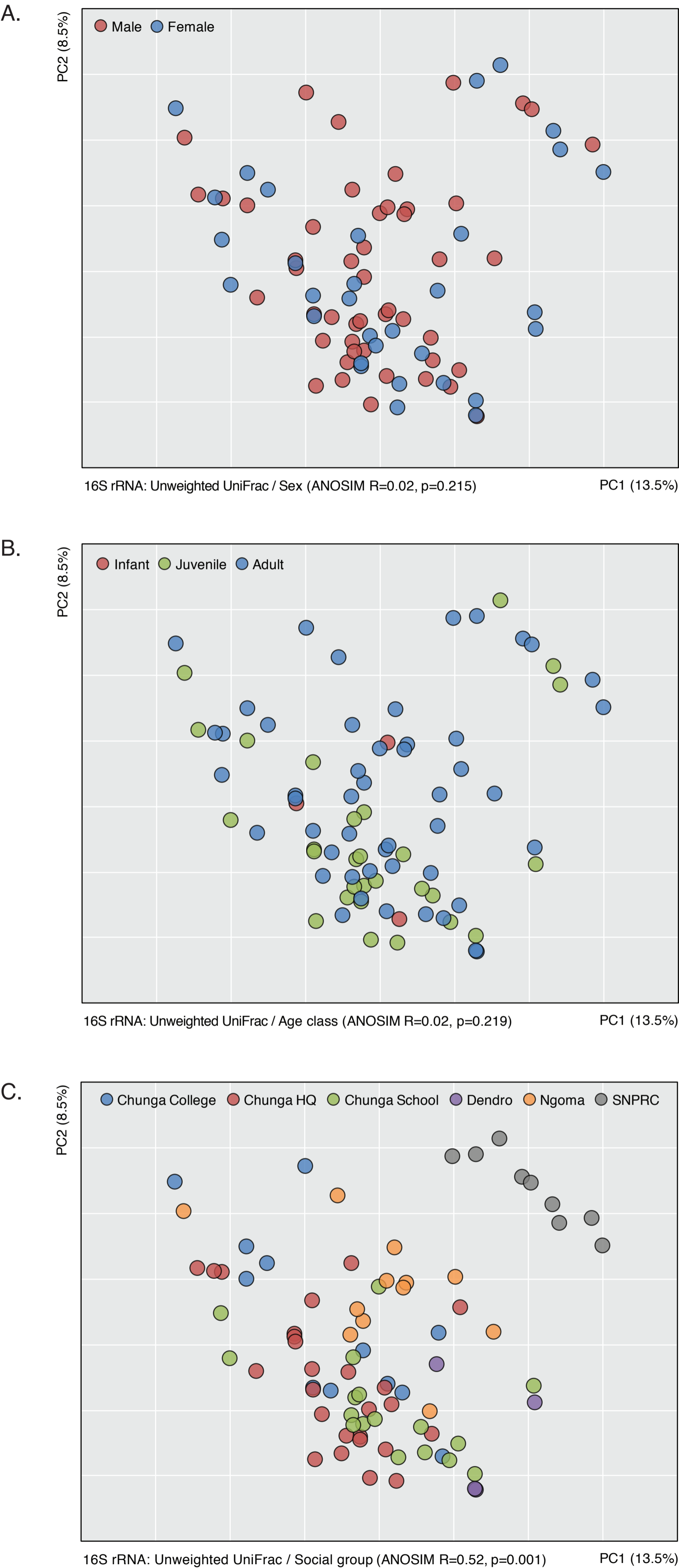

Supplement: FIG S2 [file sys003182241sf2.pdf]

Fig S3

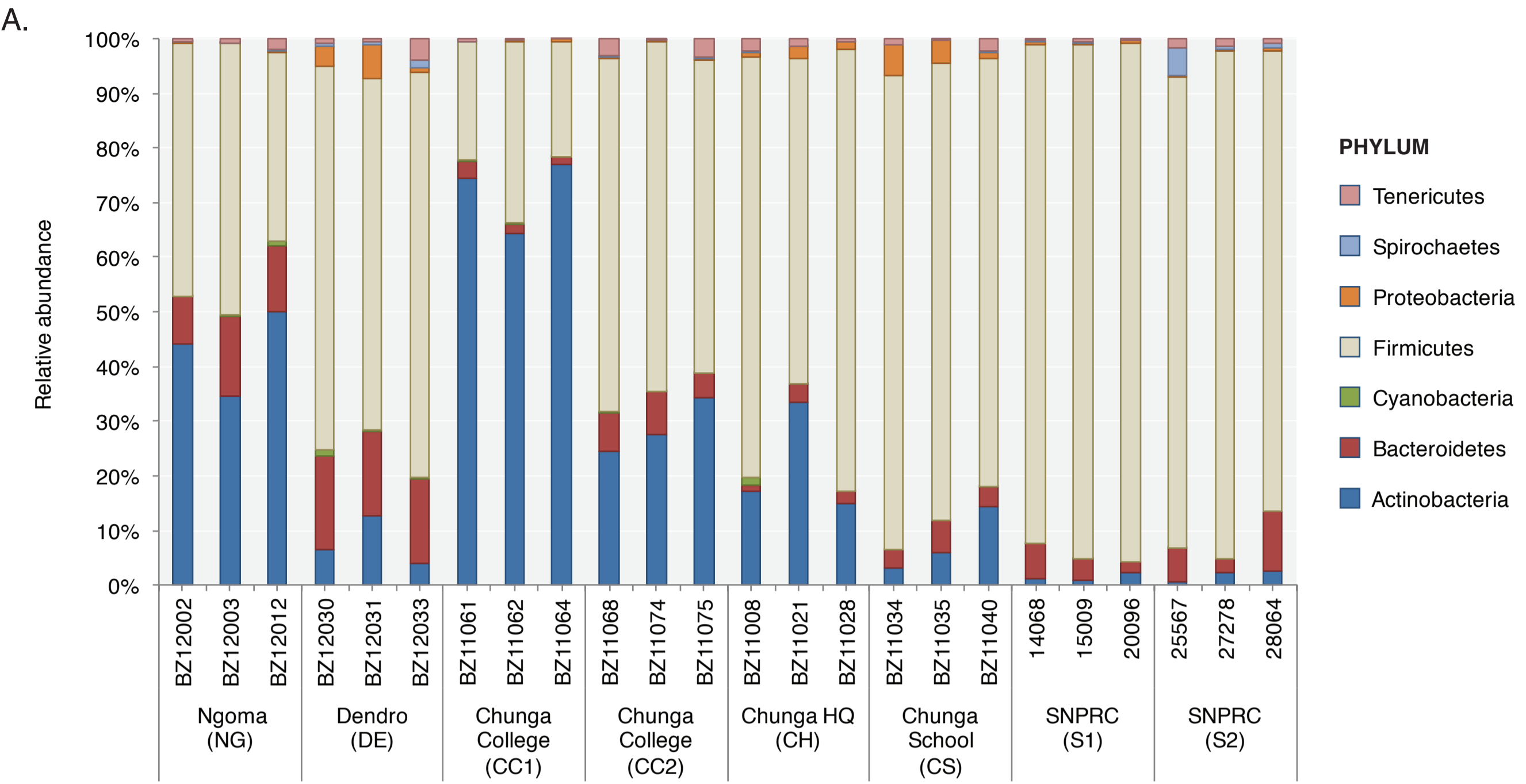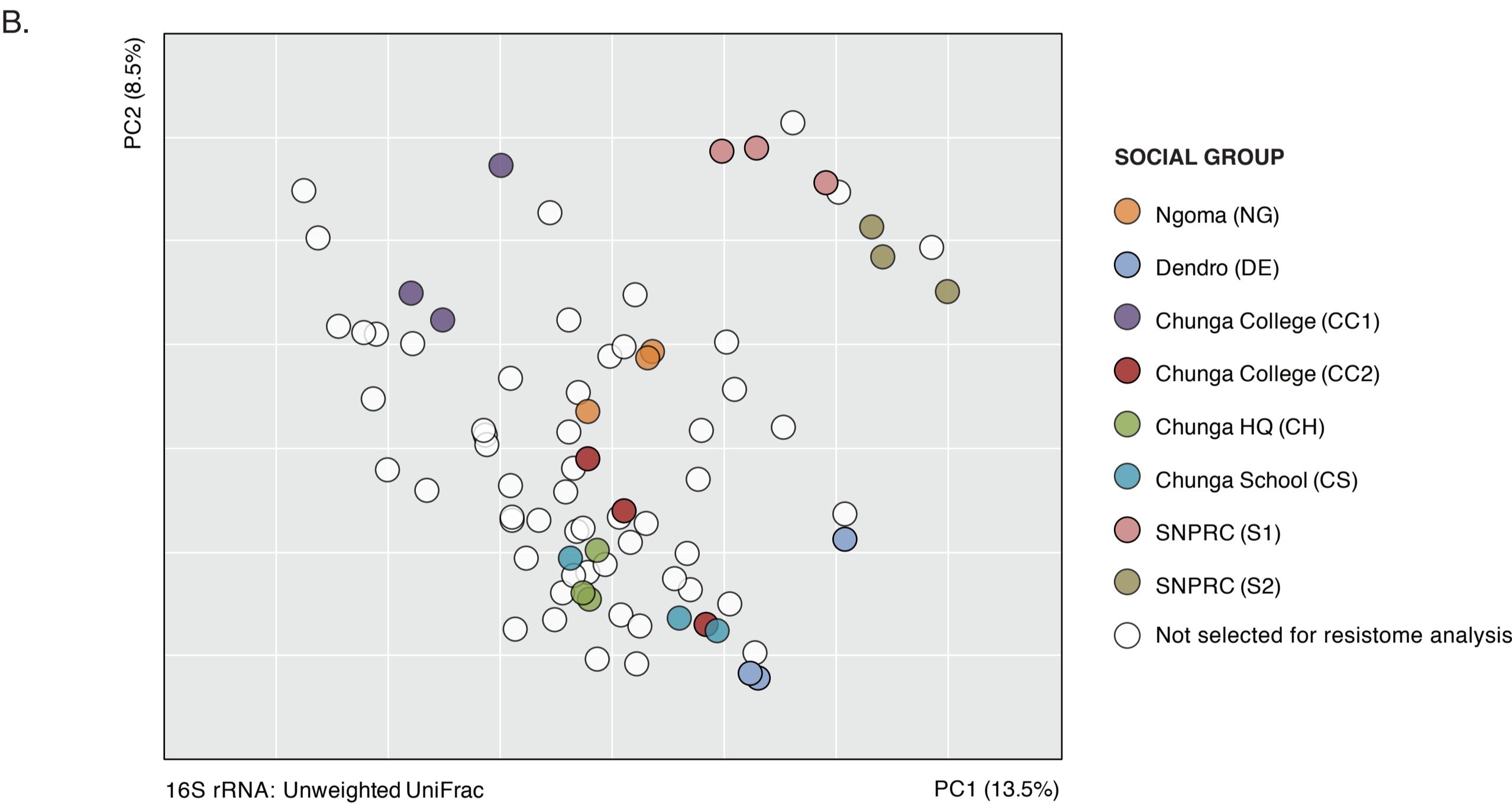

Supplement: FIG S3 [file sys003182241sf3.pdf]

Fig S4

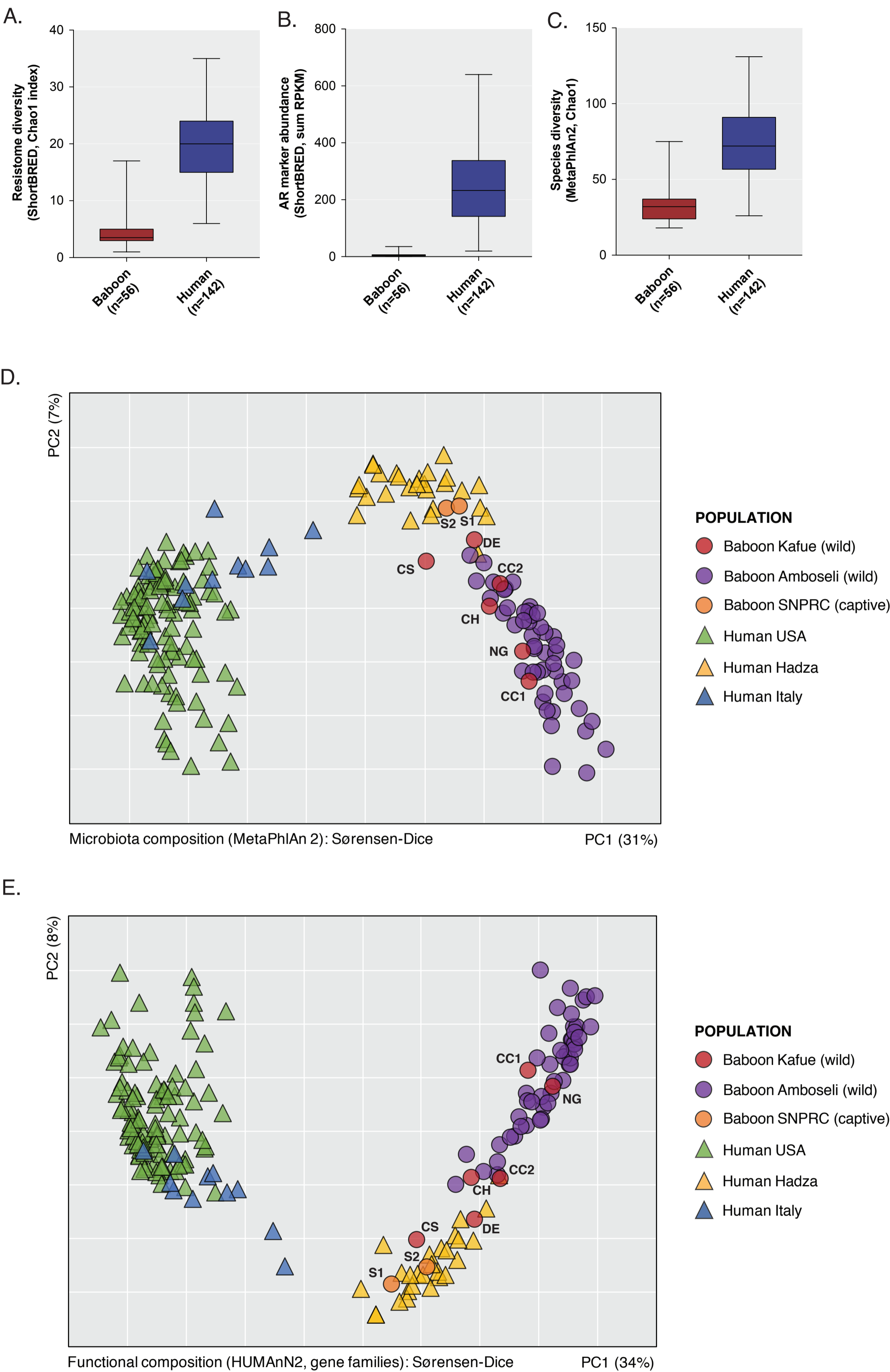

Supplement: FIG S4 [file sys003182241sf4.pdf]
